# WoVeN, the Women Veterans Network: an Innovative Peer Support Program for Women Veterans

**DOI:** 10.1007/s11606-022-07579-1

**Published:** 2022-08-30

**Authors:** Tara E. Galovski, Amy E. Street, Virginia K. McCaughey, Emma A. Archibald, Jennifer Schuster Wachen, Aimee C. Chan

**Affiliations:** 1 Women’s Health Sciences Division, National Center for PTSD, VA Boston Healthcare System, 150 South Huntington Street, Boston, MA 02130 USA; 2 Department of Psychiatry, Boston University School of Medicine, Boston, MA USA; 3 Psychology Department, Suffolk University, Boston, MA USA

## INTRODUCTION

“Being a part of WoVeN means I belong. The feeling of belonging to the military was monumental to me. And now, as a Veteran, I get to feel connected again.”

-WoVeN Member

The number of women serving in the military has grown rapidly, currently representing about 15% of the U.S. Armed Forces.^1,2^ Despite rising numbers, women Veterans are at particularly high risk for loneliness and isolation given their minority status during service as well as in civilian communities where few women share their life experiences.^3,4^ Difficulties adapting to civilian life can include mental and physical wounds of war,^5–7^ compromised relationships and family functioning,^8–10^ and challenging employment transitions,^5,8,9,11^ resulting in poor social support^5,12–14^ and compromised physical/emotional health and well-being.^3,5,11,15,16^ Isolation after separation from service,^3,4,17^ poor understanding of the unique nature of military culture within civilian society,^15,18–20^ and lack of recognition for women’s service^4,15,21^ are unique predictors of poor mental and physical health outcomes for women Veterans.

Interventions designed to mitigate effects of loneliness are gaining increasing attention^22^ and typically rely on incorporating social support into recipients’ lives. Although peer-facilitated social support interventions have been developed across populations with different areas of need,^23–25^ prior to the Women Veterans Network (WoVeN), there was no national peer support network specifically designed to meet the unique needs of women Veterans. Given the isolation and loneliness described by women Veterans, there was a substantial need for a sustainable social support network at both local and national levels that encompassed topics identified by women Veterans as central to improving quality of life using military culturally informed content developed by national experts^17,26,27^ (e.g., VA National Center for PTSD researchers) and Veteran focus groups and consultants.

### Project Goals

To answer this need, WoVeN was designed with the support of foundation funding as an innovative peer support network.^28^ The primary aims of this program are to (1) foster connections and build relationships among women Veterans in local communities and nationwide and (2) develop a collaborative network of stakeholders, agencies, and organizations that share the goal of improving the quality of life of women Veterans (e.g., VA, Veteran service organizations, women Veteran-owned businesses, Veteran interest groups).

## SETTING AND PARTICIPANTS

**Figure 1:**
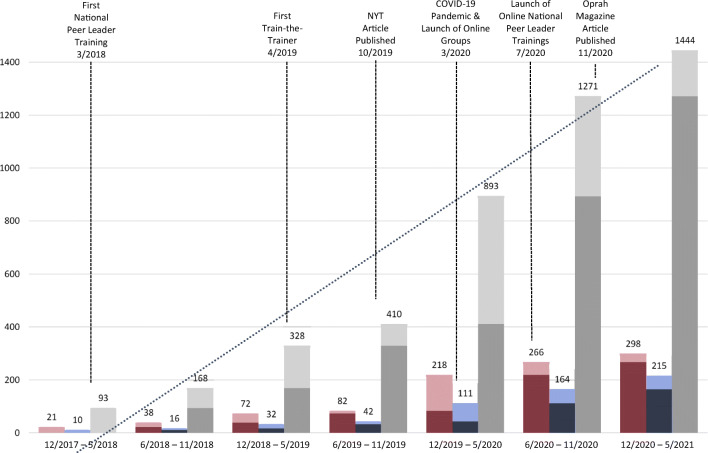
Growth of WoVeN program between December 2017 and May 2021, as measured by Peer Leaders trained, groups run, and group members enrolled.

WoVeN welcomes all women-identifying Veterans of U.S. military service, regardless of era served, branch of service, or type of discharge. WoVeN’s core component is a series of structured meetings led by vetted and trained Peer Leaders, offered locally in person and nationally via web-based meeting platforms. Since the program’s inception in 2017, WoVeN has trained 298 Peer Leaders who have led 215 groups, serving 1444 women Veterans (see Fig. 1). Beyond participating in individual groups, WoVeN offers additional opportunities for women to connect with one another and with other agencies and organizations that align with WoVeN’s mission. Over 3300 individuals have connected with WoVeN via an active social media presence, over 2100 individuals receive WoVeN’s weekly newsletter, and our program website (*www.wovenwomenvets.org*; over 40,000 visits to date) provides information for women Veterans to connect with vetted resources in important domains (e.g., healthcare, mental healthcare, employment, housing, education). WoVeN staff selects resources to be included by reviewing the resource’s website and any relevant literature and, in many cases, conducting interviews with agency staff to ensure that the partnership supports the WoVeN mission and promotes the well-being of women Veterans.

## PROGRAM DESCRIPTION

The development of WoVeN began with a literature review and focus groups conducted in three cities (Pittsburgh, Charlotte, and San Antonio) to understand the unique needs and challenges of women Veterans and Veterans’ preferences for program structure. Themes of isolation and loneliness emerged, suggesting that the primary goal of the program should be forging connections among participants. WoVeN staff developed the core curriculum of eight, 90-min sessions led by two co-Peer Leaders. The curriculum was designed to first develop, and then strengthen, relationships among members of the closed groups. Captured in the WoVeN Peer Leader Manual,^29^ meetings center around themes including Introductions, Transitions, Balance, Stress Relief, Connections, Trust, Esteem, and Reflections/Celebration. Open-ended discussion questions query both military-related and general life experiences, providing opportunities for participants to discover points of commonality as well as appreciate individual differences.

### WoVeN  Study Pilot

In August 2017, a pilot of the WoVeN program launched with a peer leader training conducted by WoVeN Directors, implementation of the WoVeN group curriculum by three pairs of women Veteran Peer Leaders in their home communities, and weekly consultation calls for implementation support and fidelity checks. Key lessons learned included the need for additional centralized support and resources (e.g., tip sheets, sharing of best practices across peer leaders, outreach materials to facilitate recruitment) and the critical need for two Peer Co-Leaders given the attrition inherent in an all-volunteer program.

### WoVeN Expansion

Following the pilot, WoVeN grew exponentially (see Fig. 1) with new Peer Leaders trained by Co-Directors via national, in-person trainings across the country. Approaches to recruitment and growth included outreach by Peer Leaders at local events, social media engagement, paid Facebook ads in targeted cities, connections with partnering organizations, invited addresses at Veteran events and professional conferences, and via popular media (e.g., National Public Radio, *New York Times*, *Oprah Magazine*). High-quality, engaging promotional materials (e.g., YouTube videos, flyers, brochures) were instrumental in the recruitment process. Investment in website development and related technology solutions was critical to automate program and communications functions that became too labor intensive to manage manually (e.g., streamlined customer relationship management system, automated email distribution).

### Train-the-Trainer Model of Dissemination

As the program grew, Peer Leader trainings conducted solely by Directors became unsustainable. Implementing a Train-the-Trainer model, wherein experienced Peer Leaders were trained to train new Peer Leaders allowed for more Peer Leaders to be trained in shorter timeframes. WoVeN staff developed a Trainers’ Manual^30^ detailing a 1-day training curriculum for selected Peer Leaders to become WoVeN National Trainers, who then train new Peer Leaders on the subsequent 2 days. The training model centers around “learning by experiencing” wherein instruction in how to lead group meetings is embedded within the administration of the core curriculum. The Train-the-Trainer model has been successful in expanding the national WoVeN footprint by training 258 Peer Leaders over 25 months compared to 40 trained in 14 months by WoVeN Directors alone (see Fig. 1).

Other innovative approaches to training include a manualized Apprentice Model of Training^19^ in which a new Peer Leader is trained by apprenticing with an experienced Peer Leader during an actual WoVeN group. Trainers could also organize smaller regional trainings following the same format as national trainings. To maintain fidelity across trainings, WoVeN Directors created a standardized training video tool^31^ focused on aspects of the program that are more difficult to teach (e.g., leadership skills, Socratic Dialogue), allowing Trainers to focus on teaching the core curriculum. Under the expanded Train-the-Trainer model, WoVeN staff provided ongoing logistical and informational support for Trainers who, in turn, provided ongoing support to Peer Leaders as they implemented groups nationwide.

### Online Delivery

Given the low base rates of women Veterans in the general population, it is not possible to populate in-person groups in every geographic location. Women in rural areas, those with extensive child/eldercare responsibilities, and those who are housebound due to disability or transportation challenges often face significant difficulties in accessing resources.^32,33^ To increase access, WoVeN staff modified the core curriculum for online delivery, piloting them in October 2019. Soon thereafter, the COVID-19 global pandemic prohibited in-person groups. In March 2020, WoVeN pivoted entirely to an online format with groups conducted via a web meeting platform. During this challenging time, many women Veterans turned to WoVeN as a critical source of support. The increased need for connection and social support during the pandemic was exemplified by Peer Leaders running groups with meetings two or three times a week in some cases. Given the reduced burdens of local recruitment, securing meeting space and transportation, the numbers of women engaging in WoVeN groups increased rapidly (see Fig. 1). To date, WoVeN has conducted 104 online groups for 538 women. Despite the success of the online modality, 19% of the women who enrolled in WoVeN immediately before or during the pandemic preferred to be put on a waitlist for an in-person group experience.

In July 2020, the online delivery of programming extended to Peer Leader trainings and a Train-the-Trainers workshop. Online trainings offered substantial benefits including cost-effectiveness, greater accessibility, and the ability to provide smaller, more frequent trainings. The approach also allowed for more vetting of and commitment building among potential Peer Leaders. Online trainings were as successful as in-person trainings with comparable retention rates of Peer Leaders trained in person (65%) and online (67%). Please see the Appendix for a visual of the WoVeN program structure.

## PROGRAM EVALUATION

Women who enroll in groups are invited to voluntarily participate in the research component of the program conducted with the human subject protection oversight of the Boston University Institutional Review Board. Research consists of 20-min electronic surveys sent prior to starting a WoVeN group, following the conclusion of group, and at 1- and 3-month follow-up intervals. As of December 2020, 669 women (46.33% of program participants) provided research data in this ongoing program. Table 1 provides baseline demographics (*N* = 669) and available program evaluation results (*N* = 176). In brief, 88.62% participants reported they would be “very likely” to refer another Veteran to WoVeN and 85.71% would participate in future WoVeN events. Among women who attended 5 or more meetings, 88.89% reported that they enjoyed group content “quite a bit or extremely” and 78.63% gained “quite a bit or extreme” value from their experience. Most participants (73.16%) reported that WoVeN had positively impacted two or more major life domains (e.g., social, recreational, work, family, religion, mental/physical health, and well-being). Self-reported levels of group enjoyment and value gained did not significantly differ between in-person and online group participants, supporting the feasibility and acceptability of leveraging online platforms for social support among women Veterans. These global reports of improvements in functioning provide indirect evidence that program goals are being met, but further evaluation of the impact of WoVeN using standardized measures is necessary. To date, WoVeN groups have been conducted in a number of public and private mental healthcare settings. Future research might explore how social support via this model might enhance clinical care.

**Table 1:** Baseline Demographics and Interim Program Evaluation Data by Group Type

Study participants (*n*)*	Full sample	Group type		
		In person	Online	
	699	363	336	
	*n* (%)	*n* (%)		
	Mean (SD)	Mean (SD)		
Race/ethnicity				
Asian	10 (1.68%)	3 (1.40%)	7 (2.47%)	
Black	215 (36.07%)	78 (36.45%)	103 (36.40%)	
White	278 (46.64%)	102 (47.66%)	131 (46.29%)	
Other	93 (15.60)	31 (14.48%)	42 (14.84%)	
Ethnicity				
Hispanic	76 (12.60%)	25 (11.52%)	37 (12.94%)	
Age	47.61 (10.42)	46.84 (9.90)	47.61 (10.76)	
Education				
High school diploma/GED	11 (1.83%)	5 (2.30%)	5 (1.75%)	
Vocational training or some college credit	118 (19.3%)	52 (23.96%)	48 (16.84%)	
Associates degree	97 (16.14%)	27 (12.44%)	49 (17.19%)	
Bachelor’s degree	188 (31.28%)	62 (28.57%)	94 (32.98%)	
Master’s, doctorate, or professional degree	187 (31.11%)	71 (32.72%)	89 (31.23%)	
Vocation				
Full-time work	195 (36.11%)	79 (39.11%)	82 (32.16%)	
Part-time work	21 (3.89%)	8 (3.96%)	12 (4.71%)	
Looking for paid work	25 (4.63%)	10 (4.95%)	11 (4.31%)	
Disabled	195 (36.11%)	60 (29.70%)	105 (41.18%)	
Other (caretaker, volunteer, student)	104 (19.26%)	45 (22.29%)	45 (17.64%)	
Military service (in years)	11.55 (7.98)	11.21 (7.82)	11.51 (8.10)	
Military branch				
Army	255 (46.03%)	84 (40.78%)	134 (50.57%)	
Marine Corps	43 (7.76%)	21 (10.19%)	17 (6.42%)	
Navy	137 (24.73%)	53 (25.73%)	61 (23.02%)	
Air Force	116 (20.94%)	48 (23.30%)	50 (18.87%)	
Coast Guard	3 (0.54%)	0	3 (1.13%)	
Military component				
Active duty	511 (92.24%)	195 (94.65%)	241 (90.95%)	
Reserves	23 (4.15%)	6 (2.92%)	15 (5.66%)	
National Guard	20 (3.61%)	5 (2.43%)	9 (3.40%)	
Military occupation specialty				
Combat arms	13 (2.38%)	6 (2.93%)	6 (2.29%)	
Combat support	180 (32.97%)	64 (31.22%)	85 (32.44%)	
Service support	353 (64.65%)	135 (65.85%)	171 (65.27%)	
Interim results for ongoing program evaluation data contributed by WoVeN group completers^†^				
	Full sample	In person	Online	
	*n* (%)	*n* (%)		
	Mean (SD)	Mean (SD)		*p*
Meeting themes: enjoyment^‡^	4.15 (0.79)	4.21 (0.73)	4.06 (0.91)	0.27
Meeting themes: value gained^‡^	3.92 (0.86)	3.95 (0.81)	3.92 (0.91)	0.87
Likelihood of sending a fellow woman Veteran to WoVeN^§^	3.83 (0.52)	3.83 (0.48)	3.83 (0.62)	0.93
Not at all	2 (1.20%)	0 (0.00%)	2 (3.85%)	N/A
A little bit	5 (2.99%)	5 (2.99%)	0 (0.00%)	N/A
Somewhat	12 (7.19%)	9 (7.83%)	3 (5.77%)	N/A
Very likely	148 (88.62%)	101 (87.83%)	47 (90.38%)	N/A
Likelihood of participating in future WoVeN events^§^	3.80 (0.51)	3.82 (0.47)	3.79 (0.60)	0.77
Not at all	1 (0.60%)	0 (0.00%)	1 (1.89%)	N/A
A little bit	6 (3.57%)	4 (3.48%)	2 (3.77%)	N/A
Somewhat	17 (10.12%)	13 (11.30%)	4 (7.55%)	N/A
Very likely	144 (85.71%)	98 (85.22%)	46 (86.79%)	N/A
	*n* (%)	*n* (%)		*χ*^2^
Positive WoVeN–related impact on life domain				
Social life	124 (74.70%)	85 (75.22%)	39 (73.58%)	0.82
Mental health and well-being	124 (75.61%)	82 (73.87%)	42 (79.25%)	0.45
Recreational activities	92 (55.09%)	68 (59.65%)	24 (45.28%)	0.08
Work or school life	78 (46.71%)	52 (45.61%)	26 (49.06%)	0.68
Physical health	64 (38.55%)	38 (33.63%)	26 (49.06%)	0.06
Family life	63 (38.18%)	42 (37.50%)	21 (39.62%)	0.79
Religious life	42 (25.30%)	29 (25.66%)	13 (24.53%)	0.88

*Totals may be < *n* = 699 due to item-level missing data

^†^Group completers were 56.88% of participants with post data; group completers participated in 5+ groups

^‡^Likert scale ranging from 1 (not at all) to 5 (extremely)

^§^Likert scale ranging from 1 (not at all) to 4 (very likely)

## DISCUSSION

### New Horizons

Since the inception of WoVeN, the most commonly asked question has been, “What’s next?” Women who complete WoVeN groups and want to continue in the program have several options including staying connected with cohorts locally, with the national community through social media, joining a new group, or training as a Peer Leader. To offer additional opportunities for engagement, staff developed 10 Alumni Group sessions, which are structurally similar to core group sessions and center around new themes such as Hope, Community, and Reintegration. WoVeN groups can continue forward as a cohort using these additional modules, or graduates from across the country can join a national Alumni Group and meet more women Veterans. To date, over 58 Alumni groups have been conducted for approximately 300 women.

In response to the often-heard sentiment that Veterans wish they had had this program when they separated from service to assist with the civilian transition, WoVeN piloted an expansion program to support women service members transitioning out of the Armed Forces. Called “Bridging ReIntegration from Dreams and Goals to Execution and Success” (BRIDGES), this expansion provides one-on-one mentorship and support during the challenging reintegration process culminating in a warm handoff to the larger WoVeN community. WoVeN women can apply to become Guides (16 women trained to date) for service members (Battle Buddies) and “give back” to this unique community in the way that only Veterans understand.

### Limitations:

This program is not without limitations. First, in a national, community-based program such as WoVeN, it is difficult to evaluate the effectiveness of specific core components given the uncontrolled research design. Information from focus groups, monthly meetings with Peer Leaders (led by National Trainers) and with National Trainers (led by WoVeN Staff), qualitative data collected in open-ended survey questions and anecdotal reports from the field supplement our empirical data to assess effectiveness and inform future directions. Second, a volunteer workforce is a strength in many ways but also means Trainers and Peer Leaders may face significant competing demands. Third, a national footprint is a lofty goal and our waitlist of 650+ women is an ongoing challenge. There are a variety of reasons for this waitlist, each requiring different strategies to address. For example, logistical barriers can be reduced by offering additional groups at a wider variety of times to increase access. For women who are hesitant to begin a group experience, WoVeN is developing additional opportunities to best meet individual needs including educational webinars, meet and greets, and informational meetings.

## Supplementary Information

ESM 1 (DOCX 896 kb)

## References

[1] National Center for Veterans Analysis and Statistics. Women veterans report: The past, present, and future of women veterans [Internet]. Washington (DC): Department of Veterans Affairs; 2017. [cited 2021 Jun 8] Figure 1a. Female Active-Duty Military Personnel: 1945-2015; p. 14. Available from:https://www.va.gov/vetdata/docs/SpecialReports/Women_Veterans_2015_Final.pdf.

[2] National Center for Veterans Analysis and Statistics. Women veterans report: The past, present, and future of women veterans [Internet]. Washington (DC): Department of Veterans Affairs; 2017. [cited 2021 Jun 8] p.72. Available from: https://www.va.gov/vetdata/docs/SpecialReports/Women_Veterans_2015_Final.pdf.

[3] Villagran M, Ledford CJ, Canzona MR (2015). Women’s health identities in the transition from military member to service veteran. J Health Commun..

[4] Demers AL (2013). From death to life: female veterans, identity negotiation, and reintegration into society. J Humanist Psychol..

[5] Street AE, Gradus JL, Giasson HL, Vogt D, Resick PA (2013). Gender differences among veterans deployed in support of the wars in Afghanistan and Iraq. J Gen Intern Med..

[6] Thomas MM, Harpaz-Rotem I, Tsai J, Southwick SM, Pietrzak RH. Mental and physical health conditions in US combat veterans: results from the National Health and Resilience in Veterans Study. Prim Care Companion CNS Disord. 2017 Jun 22;19(3): 10.4088/PCC.17m02118. 10.4088/PCC.17m02118 28657698

[7] Bonanno GA, Mancini AD, Horton JL, Powell TM, LeardMann CA, Boyko EJ, Wells TS, Hooper TI, Gackstetter GD, Smith TC (2012). Millennium Cohort Study Team. Trajectories of trauma symptoms and resilience in deployed US military service members: prospective cohort study. Br J Psychiatry..

[8] Vogt D, Smith BN, Fox AB, Amoroso T, Taverna E, Scnurr PP (2017). Consequences of PTSD for the work and family quality of life of female and male US Afghanistan and Iraq war veterans. Soc Psychiatry Psychiatr Epidemiol..

[9] Creech SK, Swift R, Zlotnick C, Taft C, Street AE (2016). Combat exposure, mental health, and relationship functioning among women veterans of the Afghanistan and Iraq Wars. J Fam Psychol..

[10] Sayer NA, Noorbaloochi S, Frazier P, Carlson K, Gravely A, Murdoch M (2010). Reintegration problems and treatment interests among Iraq and Afghanistan combat veterans receiving VA medical care. Psychiatr Serv..

[11] Nillni YI, Gradus JL, Gutner CA, Luciano MT, Shipherd JC, Street AE (2014). Deployment stressors and physical health among OEF/OIF Veterans: the role of PTSD. Health Psychol..

[12] Street AE, Vogt D, Dutra L (2009). A new generation of women veterans: stressors faced by women deployed to Iraq and Afghanistan. Clin Psychol Rev..

[13] Vogt DS, Pless AP, King LA, King DW (2005). Deployment stressors, gender, and mental health outcomes among Gulf War I veterans. J Trauma Stress..

[14] Smith BN, Wang JM, Vaughn-Coaxum RA, Di Leone BA, Vogt D (2017). The role of postdeployment social factors in linking deployment experiences and current posttraumatic stress disorder symptomatology among male and female veterans. Anxiety Stress Coping..

[15] Levander XA, Overland MK (2015). Care of women veterans. Med Clin..

[16] Teo AR, Marsh HE, Forsberg CW, Nicolaidis C, Chen JI, Newsom J, Saha S, Dobscha SK. Loneliness is closely associated with depression outcomes and suicidal ideation among military veterans in primary care. J Affect Disord. 2018 Apr 1;230:42-9. 10.1016/j.jad.2018.01.003 29407537

[17] Wilson G, Hill M, Kiernan MD (2018). Loneliness and social isolation of military veterans: systematic narrative review. Occup Med..

[18] Ainspan ND, Penk WE, editors. Returning wars' wounded, injured, and ill: A reference handbook. Westport (CT): Praeger Security International; 2008.

[19] Junger S. Tribe: On homecoming and belonging. 1st ed. Southern Pines (NC): 12 Twelve Publication Corp; 2016.

[20] Hall LK (2011). The importance of understanding military culture. Soc Work Health Care..

[21] Burkhart L, Hogan N (2015). Being a female veteran: a grounded theory of coping with transitions. Soc Work Ment Health..

[22] Cacioppo S, Grippo AJ, London S, Goossens L, Cacioppo JT (2015). Loneliness: clinical import and interventions. Perspect Psychol Sci..

[23] Barber JA, Rosenheck RA, Armstrong M, Resnick SG (2008). Monitoring the dissemination of peer support in the VA Healthcare System. Commun Ment Health J..

[24] Greden JF, Valenstein M, Spinner J (2010). Buddy-to-Buddy, a citizen soldier peer support program to counteract stigma, PTSD, depression, and suicide. Ann N Y Acad Sci..

[25] Resnick SG, Rosenheck RA (2008). Integrating peer-provided services: a quasi-experimental study of recovery orientation, confidence, and empowerment. Psychiatr Serv..

[26] Vogt D, King MW, Borowski S, Finley EP, Perkins DF, Copeland LA (2021). Identifying factors that contribute to military veterans’ post-military well-being. Appl Psychol: Health and Well-Being..

[27] Drebing CE, Reilly E, Henze KT, Kelly M, Russo A, Smolinsky J, Gorman J, Penk WE (2018). Using peer support groups to enhance community integration of veterans in transition. Psychol Serv..

[28] WoVeN Women Veterans Network [Internet]. Women Veterans Network; 2015 [cited 2021 Jun 8]. Available from: https://www.wovenwomenvets.org/.

[29] Galovski TE, Street AE, McCaughey VK, Corchado C, Rosado T, Wachen JS. WoVeN Women Veterans Network Peer Leader Manual December 2020. Boston, 2020. 174 p.

[30] Galovski TE, McCaughey VK, Archibald EA, Wachen JS, Sawdy M, Street AE. WoVeN women Veterans network trainer’s manual April 2021. Boston, 2021. 172p.

[31] WoVeN. Peer Leader Training: Introduction to WoVeN [Internet]. YouTube. 2019 [cited 2021 Jun 9]; Available from https://www.youtube.com/watch?v=g74-IQ75qR4.

[32] Washington DL, Bean-Mayberry B, Riopelle D, Yano EM (2011). Access to care for women veterans: delayed healthcare and unmet need. J Gen Intern Med..

[33] Mankowski M, Everett JE (2016). Women service members, veterans, and their families: what we know now. Nurse Educ Today..

