# Supplementary material for: WoVeN, the Women Veterans Network: an Innovative Peer Support Program for Women Veterans
Source: J Gen Intern Med. 2022 Aug 30;37(Suppl 3):842–7. doi: 10.1007/s11606-022-07579-1 (PMC9427174; doi:10.1007/s11606-022-07579-1)
Supplement: Supplementary file 1 — (DOCX 896 kb) [file 11606_2022_7579_MOESM1_ESM.docx]

Appendix A

WoVeN Program Structure


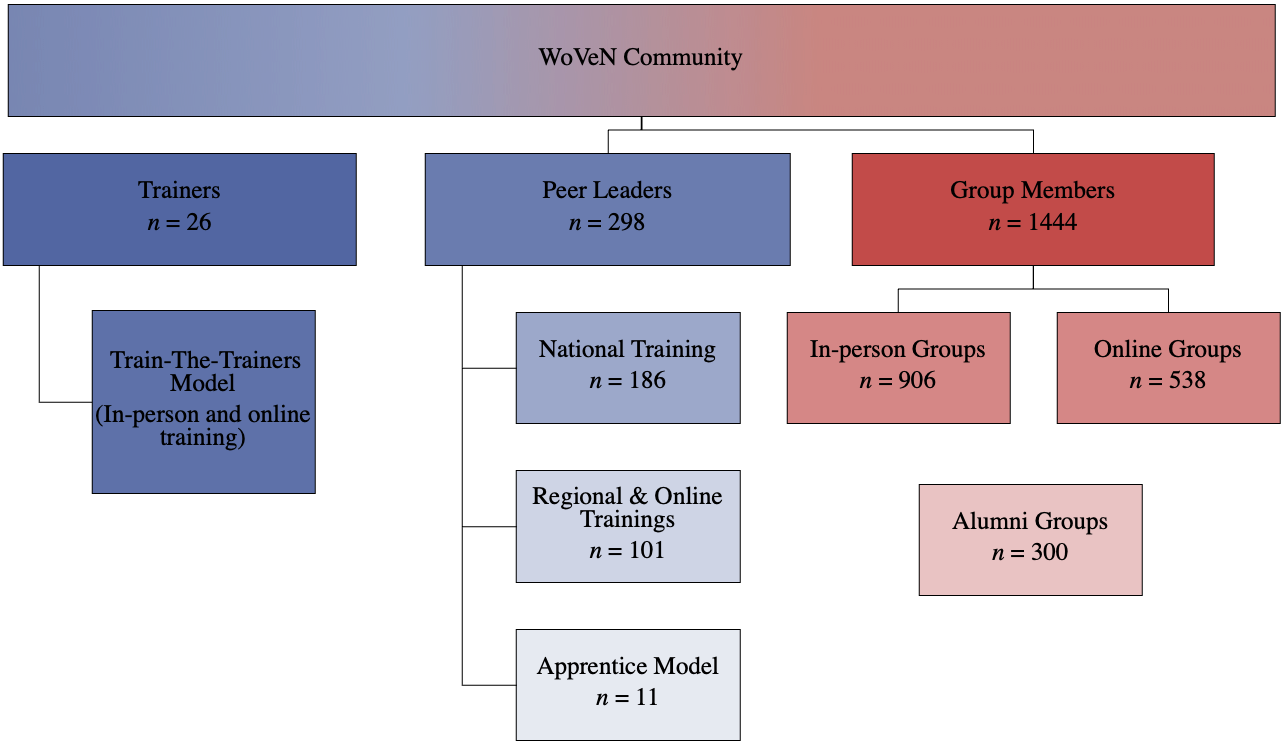

 *Note.* Dotted lines with arrowheads indicate that a subset of the preceding group(s) will go on to pursue this opportunity.
